# A novel air-dried multiplex high-resolution melt assay for the detection of extended-spectrum β-lactamase and carbapenemase genes

**DOI:** 10.1016/j.jgar.2021.08.006

**Published:** 2021-12

**Authors:** Ana I. Cubas-Atienzar, Christopher T. Williams, Abhilasha Karkey, Sabina Dongol, Manandhar Sulochana, Shrestha Rajendra, Glyn Hobbs, Katie Evans, Patrick Musicha, Nicholas Feasey, Luis E. Cuevas, Emily R. Adams, Thomas Edwards

**Affiliations:** a Centre for Drugs and Diagnostics, Liverpool School of Tropical Medicine, Liverpool, UK; b Oxford Clinical Research Unit, Patan Academy of Health Sciences, Kathmandu, Nepal; c Liverpool John Moores University, Liverpool, UK; d Wellcome Sanger Institute, Cambridge, UK; e Malawi–Liverpool–Wellcome Trust Clinical Research Programme, Blantyre, Malawi

**Keywords:** Antimicrobial resistance, Extended-spectrum β-lactamase, ESBL, Carbapenemase, High-resolution melting, Molecular diagnostics

## Abstract

**Objectives:**

This study aimed to develop and evaluate a novel air-dried high-resolution melt (HRM) assay to detect eight major extended-spectrum β-lactamase (ESBL) (*bla*_SHV_ and *bla*_CTX-M_ groups 1 and 9) and carbapenemase (*bla*_NDM_, *bla*_IMP_, *bla*_KPC_, *bla*_VIM_ and *bla*_OXA-48-like_) genes that confer resistance to cephalosporins and carbapenems.

**Methods:**

The assay was evaluated using 439 DNA samples extracted from bacterial isolates from Nepal, Malawi and the UK and 390 clinical isolates from Nepal with known antimicrobial susceptibility. Assay reproducibility was evaluated across five different real-time quantitative PCR (qPCR) instruments [Rotor-Gene® Q, QuantStudio^TM^ 5, CFX96, LightCycler® 480 and Magnetic Induction Cycler (Mic)]. Assay stability was also assessed under different storage temperatures (6.2 ± 0.9°C, 20.4 ± 0.7°C and 29.7 ± 1.4°C) at six time points over 8 months.

**Results:**

The sensitivity and specificity (with 95% confidence intervals) for detecting ESBL and carbapenemase genes was 94.7% (92.5–96.5%) and 99.2% (98.8–99.5%) compared with the reference gel-based PCR and sequencing and 98.3% (97.0–99.3%) and 98.5% (98.0–98.9%) compared with the original HRM wet PCR mix format. Overall agreement was 91.1% (90.0–92.9%) when predicting phenotypic resistance to cefotaxime and meropenem among Enterobacteriaceae isolates. We observed almost perfect inter-machine reproducibility of the air-dried HRM assay, and no loss of sensitivity occurred under all storage conditions and time points.

**Conclusion:**

We present a ready-to-use air-dried HRM PCR assay that offers an easy, thermostable, fast and accurate tool for the detection of ESBL and carbapenemase genes in DNA samples to improve antimicrobial resistance detection.

## Highlights

• Development and evaluation of a novel air-dried HRM assay to detect eight major ESBL and carbapenemase genes.
• Sensitivity and specificity of the air-dried HRM assay versus the reference molecular tests was 94.7–98.3% and 98.5–99.2%.
• Assay had overall agreement of 91.1% in predicting phenotypic resistance to cefotaxime and meropenem in Enterobacteriaceae.
• Cross-platform validation showed almost perfect reproducibility in five different qPCR platforms.
• No loss of sensitivity was observed after 8 months of storage at room, refrigerator and oven temperatures.

## 1 Introduction

Antimicrobial resistance (AMR) is a major cause of death worldwide and the development of new antibiotics is considered a public-health priority [1]. An estimated 700 000 deaths are attributable to AMR globally each year, and this number is predicted to rise to 10 million by 2050 [2]. Identification of AMR is typically by culture-based phenotypic antimicrobial susceptibility testing, which requires incubation, from the primary sample, for 48–96 h. As clinical management decisions are often taken rapidly, the lack of timeliness of antimicrobial susceptibility testing leads to empirical treatment, which is often inappropriate [3,4]. First-line or broad-spectrum antibiotics are often used in large doses to ensure their efficacy against the suspected but unknown aetiological pathogens [5,6]. Empirical treatment facilitates the emergence of AMR, increases the duration of hospitalisation, damages the patient microbiota and increases the cost of therapy [7], [8], [9]. Rapid identification of AMR can enable targeted usage of antibiotics, improved patient outcomes and antimicrobial stewardship [4,6,10,11].

The most common mechanism of drug resistance in Gram-negative bacteria is the production of β-lactamases, including extended-spectrum β-lactamases (ESBLs) and carbapenemases [12], which confer resistance to β-lactam antibiotics. PCR-based detection of ESBL and carbapenemase genes provides a faster detection of AMR than phenotypic methods, which might in turn generate more timely information for treatment decisions [13,14]. Whist molecular methods for the detection and characterisation of AMR genes are becoming increasingly established, with good agreement with phenotypic methods and producing faster results [15], [16], [17], their use in clinical settings is hampered by the high degree of multiplexing needed due to the many genes involved in an antimicrobial-resistant phenotype. Additionally, PCR requires a cold chain to maintain the integrity of reagents as well as equipment and trained staff, which are often unavailable in low- and middle-income countries (LMICs). One approach that could facilitate the implementation of PCR assays in LMICs would be to provide the PCR primers, *Taq* enzyme and buffer components dry in the PCR vessels. This process eliminates the need for a cold chain and simplifies preparation, as only the addition of nuclease-free water and the DNA template is needed to resuspend the PCR reagents [18], [19], [20]. Typically, this process would be done by lyophilisation of the reagents. Lyophilisation, also called freeze-drying, is the process of the removal of water from a product by volatilisation and desorption to increase the lifespan of a product. However, lyophilisation is costly and requires the addition of excipients, such as cryoprotectants and bulking agents [19,21]. There are commercially available ready-to-use thermostable PCR kits for the detection of AMR genes, such as GeneXpert® CarbaR (Cepheid, USA) and FilmArray^TM^ Blood Culture Identification (BioFire, USA), however these are expensive and require proprietary equipment, making them difficult to use routinely and difficult to implement in some low-resource settings [20].

Here we report the development and validation of an air-dried high-resolution melt (HRM) PCR mix to detect the most frequent ESBL and carbapenemase genes based on a previously validated in-house HRM PCR assay [22].

## 2 Methods

### 2.1 Air-dried high-resolution melt assay optimisation

We adapted an in-house 9-plex HRM PCR [22] into a dry format to detect three major ESBL genes (*bla*_SHV_ and *bla*_CTX-M_ groups 1 and 9) and five carbapenemase genes (*bla*_NDM_, *bla*_IMP_, *bla*_KPC_, *bla*_VIM_ and *bla*_OXA-48-like_). For the dry-out process, AmpDRY™ (Biofortuna, UK) was used, which is a PCR reaction mix that allows direct air-drying of the whole reaction including primers and reporter molecules and removes the need for lyophilisation systems and reagents. The composition of each HRM reaction included a mixture of 1 × EvaGreen® dye (Biotium, Canada), primers for detecting ESBL groups and carbapenemase genes [22], the proprietary air-drying PCR buffer AmpDRY™ (Biofortuna, UK) and PCR-grade water to a final volume of 6.25 μL. The reaction mixture was added into each of the wells of a 96-well PCR plate (Starlab, Germany) and was dried in an oven-drier (ElextriQ, UK) at 35°C for 17 h. PCR was performed by adding 2.5 μL of bacterial DNA and 500 mM betaine (Sigma-Aldrich, UK) in PCR-grade water to each PCR well containing the dried reagents for a final reaction volume of 12.5 μL. When plates were not compatible with the thermocycler used (Rotor-Gene® Q), PCR plates were briefly centrifuged before PCR amplification and the mixture was transferred to the appropriate reaction vessels (Rotor-Gene® Q strip tubes). The optimised PCR amplification protocol consisted of an initial incubation step at 80°C for 15 min, followed by 30 cycles of denaturation at 95°C for 10 s, annealing at 66°C for 60 s and elongation at 72°C for 10 s, monitoring the fluorescence in the FAM/SYBR channel. HRM analysis was carried out over a temperature range of 75°C to 95°C taking a reading in the HRM/SYBR channel every 0.1°C, with a 2-s stabilisation between each step. Peak calling was automated and indicated by a peak at the predictive melting temperature (*T*_m_) of the target visualised as the negative first derivative of the melting curve in the Rotor-Gene® Q software. The Rotor-Gene® Q (QIAGEN, UK) was used for all the experiments except where stated otherwise. Optimal conditions of the assay were achieved by titration of individual reaction components and optimisation of amplification conditions and drying time. The original primer mix and their concentrations were as described previously [22], except that *bla*_TEM_ was removed as it is ubiquitous in *Escherichia coli* and the most common variants are narrow spectrum.

### 2.2 Stored bacterial DNA and reference molecular tests

A panel of 439 DNA samples from well documented multidrug-resistant bacterial isolates from Nepal (*n* = 293), the UK (*n* = 103) and Malawi (*n* = 43) was used to optimise and evaluate the air-dried HRM assay.

Bacterial DNA from Nepal comprised isolates collected from 2012–2016 at Patan Hospital, Kathmandu, and included strains of *E. coli* (*n* = 112), *Acinetobacter* spp*.* (*n* = 72), *Klebsiella pneumoniae* (*n* = 54), *Enterobacter* spp*.* (*n* = 32), *Pseudomonas aeruginosa* (*n* = 20), *Proteus* spp. (*n* = 1), *Providencia rettgeri* (*n* = 1) and *Serratia rubidaea* (*n* = 1). Isolates were collected during routine diagnostic testing from clinical samples.

Bacterial DNA from Malawi comprised isolates collected between 1996–2012 at Queen Elizabeth Central Hospital, Blantyre, during routine diagnostic testing and comprised *E. coli* (*n* = 25) and *K. pneumoniae* (*n* = 18). Collection of isolates was approved by the University of Malawi College of Medicine Research and Ethics Committee (COMREC), Blantyre, under study number P.08/14/1614.

Bacterial DNA from the UK comprised isolates collected between 2012–2017 from the UK National Health Service hospitals and included *E. coli* (*n* = 40), *K. pneumoniae* (*n* = 27), *Klebsiella aerogenes* (*n* = 12), *Enterobacter cloacae* (*n* = 10), *Citrobacter freundii* (*n* = 4), *P. aeruginosa* (*n* = 4), *Morganella morganii* (*n* = 2) and *K. oxytoca* (*n* = 1). The species of three isolates could not be determined.

Further details of all isolates in the sample collection are available in the Supplementary materials.

DNA from the Nepal and Malawi isolates was extracted using the boilate [23] method, and isolates from the UK were extracted using a DNeasy Blood and Tissue Kit (QIAGEN). Isolates sourced in the UK and Nepal were screened for ESBL and carbapenemase markers using reference gel-based PCR published protocols [13,14] and the air-dried HRM assay. The reference PCR reaction mix was performed using DreamTaq PCR Reaction Mix (Thermo Fisher, UK), 2.5 μL of DNA and nuclease-free water to a final volume of 12.5 μL. PCR amplification was visualised with PicoGreen^TM^ (Life Technologies, USA) staining on a 1% TBE (Tris–borate–EDTA) gel with 1–2% of agarose depending on the fragment size to resolve. This reference gel-based PCR was not performed with the Malawian isolates as next-generation sequencing data were available from previous studies [8,22]. In addition, the 439 isolates were screened using the in-house 9-plex HRM PCR assay originally developed in our laboratory [22] using the commercially available Type-it® HRM Kit (QIAGEN).

### 2.3 Bacterial strains for phenotype prediction evaluation in Nepal

A set of 390 Gram-negative bacteria with known phenotypes were chosen based on their resistance profile from a collection of characterised clinical isolates banked at Patan Hospital in Nepal. Bacterial phenotypes were determined by the disk diffusion method following Clinical and Laboratory Standards Institute (CLSI) guidelines. Banked isolates were selected based on their resistance to meropenem (37%) and cefotaxime (85%) and were resuscitated on MacConkey agar (Thermo Fisher Scientific, USA) and DNA was extracted by a boiling lysis method as described previously [23]. Intermediate phenotypic profiles were not selected for the study. To evaluate the agreement between the phenotype and HRM result (genotype), isolates positive for any (one or more) ESBL groups and carbapenemase genes were considered resistant to cefotaxime, and isolates positive for any (one or more) carbapenemase genes were considered resistant to meropenem.

Isolates included strains of *E. coli* (*n* = 72), *K. pneumoniae* (*n* = 107), *Acinetobacter* spp. (*n* = 76), *Enterobacter* (*n* = 63), *Salmonella* Typhi (*n* = 25), *K. oxytoca* (*n* = 16), *P. aeruginosa* (*n* = 13), *Salmonella* Paratyphi (*n* = 7), *M. morganii* (*n* = 3), *C. freundii* (*n* = 2), *Serratia* spp. (*n* = 3), *Proteus* spp. (*n* = 2) and *P. rettgeri* (*n* = 1).

### 2.4 Limit of detection (LOD)

The LOD of the air-dried assay was evaluated for the ESBL genes *bla*_CTX-M-1_ and *bla*_SHV_ using one *E. coli* isolate positive for *bla*_CTX-M-1_ (isolate 1), one *K. pneumoniae* positive for *bla*_SHV_ (isolate 2) and one *K. pneumoniae* isolate harbouring both genes *bla*_CTX-M-1_ and *bla*_SHV_ (isolate 3) to estimate the LOD in isolates co-producing multiple genes. The LOD was performed following a published protocol [24]. Briefly, a single colony of each isolate was incubated at 37°C for 3 h in 5 mL of Luria–Bertani (LB) broth (Thermo Fisher Scientific, UK). Cultures were then sequentially diluted 1:10 in LB broth and 10 μL of each dilution was plated in triplicate on LB agar. The plates were then incubated overnight at 37°C and colonies were counted to quantify the CFU/mL in the suspension. Two aliquots of 200 μL of each of the suspensions were taken and processed following two extraction methodologies: DNeasy Blood and Tissue Kit (QIAGEN) and the boilate technique. DNA samples for each dilution series were tested in triplicate using the HRM assay. The LOD was defined as the lowest concentration at which the AMR genes were detected in all three replicates.

### 2.5 Cross-platform validation

To evaluate the compatibility of the air-dried HRM assay in a wide range of platforms, a set of 94 samples comprising all of the resistance genes were tested using different real-time quantitative PCR (qPCR) systems, including the Rotor-Gene® Q, QuantStudio^TM^ 5 (Thermo Fisher, USA), CFX96 (Bio-Rad, USA), LightCycler® 480 (Roche Life Sciences, Germany) and Magnetic Induction Cycler (Mic) (Bio Molecular Systems, Australia). Amplification of the markers was assessed together with changes in *T*_m_ between platforms.

### 2.6 Evaluation of stability upon storage at different temperatures

Stability of the air-dried HRM assay was evaluated over time under different storage temperatures. A set of 89 samples comprising all of the markers and isolates 1–3 at the dilution of the LOD and previous dilution were tested with plates stored at different conditions. One PCR plate with the dried reaction mix was stored for each of the following periods of time: 1 week (T1); 2 weeks (T2); 1 month (T3); 3 months (T4); and 8 months (T5). Assay stability was assessed upon storage in a refrigerator (5°C), at room temperature (20°C) and in an oven (30°C). PCR plates were sealed with foil adhesive film and were individually wrapped in heat-sealed aluminium foil-laminated pouches containing one desiccant sachet (Merck, USA). Temperature and humidity were recorded weekly.

### 2.7 Data analysis

Statistical analysis was performed with IBM SPSS Statistics v.19 (IBM Corp., Armonk, NY, USA). The outcome of all tests was labelled as 0 when negative or 1 when positive. The level of agreement between tests was determined using Cohen's kappa (κ). κ coefficients with values between 0–0.20, 0.21–0.39, 0.40–0.59, 0.60–0.79, 0.80–0.90 and 0.91–1 were interpreted as no agreement or minimal, weak, moderate, strong and almost perfect agreement, respectively [25]. The statistical significance of differences in *T*_m_ between platforms was measured using one-way analysis of variance (ANOVA), and differences in peak height between different storage conditions were measured using one-way ANOVA with Tukey's test for post-hoc analysis. Statistical significance was set at a *P*-value of <0.05.

## 3 Results

### 3.1 Air-dried high-resolution melt assay evaluation using banked DNA

**Fig. 1:**
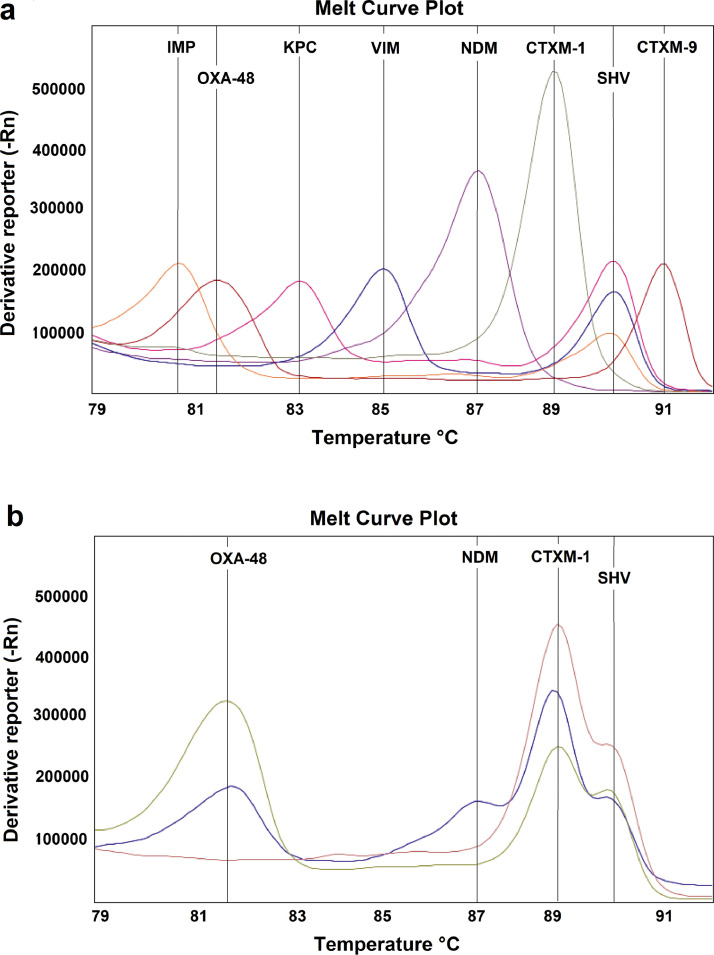
Melt curve profile of the air-dried high-resolution melt (HRM) assay showing (a) the panel comprising the eight markers, with some of the isolates also being co-producers and (b) detail of the simultaneous detection of two (pink), three (green) and four (blue) genes in isolates co-producing extended-spectrum β-lactamases (ESBLs) and carbapenemases. (a) Orange, *Klebsiella pneumoniae* harbouring *bla*_SHV_ and *bla*_IMP_ genes; red, *Escherichia coli* harbouring *bla*_OXA-48-like_ and *bla*_CTX-M-9_ genes; pink, *K. pneumoniae* harbouring *bla*_SHV_ and *bla*_KPC_ genes; blue, *K. pneumoniae* harbouring *bla*_SHV_ and *bla*_VIM_ genes; purple, *Acinetobacter* spp*.* harbouring the *bla*_NDM_ gene; and grey, *Pseudomonas aeruginosa* harbouring the *bla*_IMP_ gene. (b) Pink, *K. pneumoniae* harbouring *bla*_SHV_ and *bla*_CTX-M-1_ genes; green, *K. pneumoniae* harbouring *bla*_OXA-48-like_, *bla*_SHV_ and *bla*_CTX-M-1_ genes; and blue, *K. pneumoniae* harbouring *bla*_OXA-48-like_, *bla*_NDM_, *bla*_SHV_ and *bla*_CTX-M-1_ genes.

The air-dried HRM assay was capable of identifying the eight markers, each of which was characterised by the presence of a single peak at the expected *T*_m_ (Fig. 1a). The assay was also able to identify co-producers of up to four AMR markers (Fig. 1b). There was no overlap between adjacent peaks, with a minimum separation of peak *T*_m_ of 0.8°C allowing easy identification of multiple genes within the same sample.

Measures of diagnostic accuracy and agreement of the air-dried HRM assay for detecting individual genes compared with the reference tests are detailed in Table 1 (PCR and whole-genome sequencing) and Table 2 (original 9-plex HRM assay). The overall sensitivity and specificity of the air-dried HRM assay for all genetic markers in comparison with the reference gel-based PCR and sequencing were 94.7% [95% confidence interval (CI) 92.5–96.5%] and 99.2% (95% CI 98.8–99.5%) and in comparison with the original 9-plex HRM PCR assay [22] were 98.3% (95% CI 97.0–99.3%) and 98.5% (95% CI 98.0–98.9%). Compared with the reference gel-based PCR and sequencing, the air-dried HRM assay had almost perfect agreement (κ = 0.94–1.00) for the ESBL *bla*_CTX-M_ group and carbapenemase markers and strong agreement (κ = 0.81) for *bla*_SHV_. The *bla*_SHV_ gene was often (77/102) found in co-producers of multiple genes and the sensitivity of *bla*_SHV_ was lower in isolates carrying two (76.7%) and three (59.3%) genes compared with single producers of *bla*_SHV_ (92.6%). The air-dried HRM assay was 100% and 99.3–99.7% sensitive for detecting carbapenemase co-producer isolates in comparison with the reference gel-based/sequencing and original 9-plex HRM PCR assay, respectively.

**Table 1:** Sensitivity, specificity, accuracy and agreement (κ) of the air-dried high-resolution melt (HRM) assay for detecting individual genes compared with the reference PCR and whole-genome sequencing (WGS)

	Reference PCR/WGS		Sensitivity (%) (95% CI)	Specificity (%) (95% CI)	Accuracy (%) (95% CI)	κ
	Positive	Negative				
*bla*_CTX-M-1_			99.2 (97.7–100)	94.9 (91.7–97.5)	97.3 (95.3–98.6)	0.94
Positive	242	10				
Negative	2	185				
*bla*_CTX-M-9_			100 (76.8–100)	99.5 (98.5–99.9)	99.8 (98.7–99.9)	0.96
Positive	14	1				
Negative	0	424				
*bla*_SHV_			79.7 (71.3–86.5)	97.7 (95.5–99.1)	92.9 (89.9–95)	0.81
Positive	94	7				
Negative	24	314				
*bla*_NDM_			99.1 (95.2–99.9)	99.1 (97.3–99.9)	99.1 (97.7–99.8)	0.98
Positive	112	3				
Negative	1	323				
*bla*_IMP_			100 (15.8–100)	100 (99.2–100)	100 (99.2–100)	1.00
Positive	2	0				
Negative	0	437				
*bla*_KPC_			100 (63.1–100)	100 (99.2–100)	100 (99.2–100)	1.00
Positive	8	0				
Negative	0	431				
*bla*_OXA-48-like_			92.9 (66.1–99.8)	100 (99.2–100)	99.8 (98.8–100)	0.96
Positive	13	0				
Negative	1	425				
*bla*_VIM_			100 (80.5–100)	99.8 (98.7–99.9)	99.7 (98.7–99.9)	0.97
Positive	17	1				
Negative	0	421				

CI, confidence interval.

**Table 2:** Sensitivity, specificity, accuracy and agreement (κ) of the air-dried high-resolution melt (HRM) assay for detecting individual genes compared with the original 9-plex HRM assay [22] using Type-it® HRM buffer (QIAGEN)

	9-plex HRM		Sensitivity (%) (95% CI)	Specificity (%) (95% CI)	Accuracy (%) (95% CI)	κ
	Positive	Negative				
*bla*_CTX-M-1_			99.2 (97.7–100)	93.1 (88.4–96.3)	96.6 (94.4–98.1)	0.93
Positive	237	13				
Negative	2	187				
*bla*_CTX-M-9_			100 (76.8–100)	99.8 (98.7–99.9)	99.8 (98.7–99.9)	0.96
Positive	14	1				
Negative	0	424				
*bla*_SHV_			97.7 (91.7–99.7)	95.5 (91.9–99.7)	95.9 (93.6–97.5)	0.88
Positive	84	16				
Negative	2	337				
*bla*_NDM_			97.3 (92.1–99.4)	97.6 (95.3–99.0)	97.5 (95.6–98.9)	0.93
Positive	106	8				
Negative	3	322				
*bla*_IMP_			100 (2.5–100)	99.8 (98.7–99.9)	99.8 (98.7–99.9)	0.67
Positive	1	1				
Negative	0	437				
*bla*_KPC_			100 (63.1–100)	100 (99.2–100)	100 (99.2–100)	1.00
Positive	8	0				
Negative	0	431				
*bla*_OXA-48-like_			100 (73.5–100)	99.8 (98.7–100)	99.8 (98.8–100)	0.96
Positive	12	1				
Negative	0	426				
*bla*_VIM_			100 (75.3–100)	98.6 (96.9–99.5)	98.7 (98.4–99.9)	0.85
Positive	13	5				
Negative	0	421				

CI, confidence interval.

### 3.2 Bacterial strains for phenotype prediction evaluation from Nepal

The overall percentage agreement of the air-dried HRM result and phenotype was 92.4% (95% CI 89.9–94.4%) for Enterobacteriaceae isolates and 57.1% (95% CI 49.6–64.4%) for non-Enterobacteriaceae isolates. The air-dried HRM assay had strong agreement with the phenotype (κ = 0.845) among Enterobacteriaceae isolates with a sensitivity in predicting resistance to cefotaxime of 92.1% (95% CI 88.0–95.1%) and in predicting resistance to carbapenems of 84.2% (95% CI 75.3–90.9%). However, the phenotype was poorly predicted among non-Enterobacteriaceae isolates using the air-dried HRM assay (Table 3). Sensitivity to meropenem was stratified by gene detected to investigate whether the presence a particular carbapenemase gene was associated with false positivity, as the carriage of *bla*_OXA-48-like_ genes does not always confer resistance to meropenem [26]. In this study, the presence of *bla*_OXA-48-like_ or any other carbapenemase gene was not associated with an increase of false-positive rate in meropenem-sensitive isolates (χ^2^ test, *P* > 0.05).

**Table 3:** Sensitivity, specificity, accuracy and agreement (κ) of the air-dried high-resolution melt (HRM) assay compared with the phenotype in isolates from Nepal

	Phenotype		Sensitivity (%) (95% CI)	Specificity (%) (95% CI)	Accuracy (%) (95% CI)	κ
Enterobacteriaceae						
HRM Carb	MEM-R	MEM-S				
Positive	80	6	84.2 (75.3–90.9)	97.1 (93.8–98.2)	93.2 (89.5–95.6)	0.834
Negative	15	200				
HRM Carb/ESBL	CTX-R	CTX-S				
Positive	232	5	92.1 (88.0–95.1)	89.80 (77.8–96.6)	91.69 (88.0–94.6)	0.729
Negative	20	44				
Non-Enterobacteriaceae						
HRM Carb	MEM-R	MEM-S				
Positive	23	5	46.0 (31.8–60.7)	87.8 (73.8–95.9)	64.8 (54.1–74.6)	0.313
Negative	27	36				
HRM Carb/ESBL	CTX-R	CTX-S				
Positive	42	4	50.0 (38.9–61.1)	42.9 (9.9–81.6)	49.5 (38.8–60.1)	0.020
Negative	42	3				

CI, confidence interval; MEM-R, meropenem-resistant; MEM-S, meropenem-susceptible; ESBL, extended-spectrum β-lactamase; CTX-R, cefotaxime-resistant; CTX-S, cefotaxime-susceptible.

### 3.3 Cross-platform validation

Almost perfect reproducibility was obtained for all instruments. A cut-off was established for each instrument by evaluating five threshold values set as 20%, 10%, 7.5%, 5% and 3% of the fluorescence of the highest peak. The optimal cut-off for the Rotor-Gene® Q, QuantStudio^TM^ 5 and Mic was 5% of the fluoresce of the highest peak, and for CFX96 and LightCycler® 480 it was 10%. These cut-offs produced almost perfect agreement with the reference tests (κ = 0.935).

**Fig. 2:**
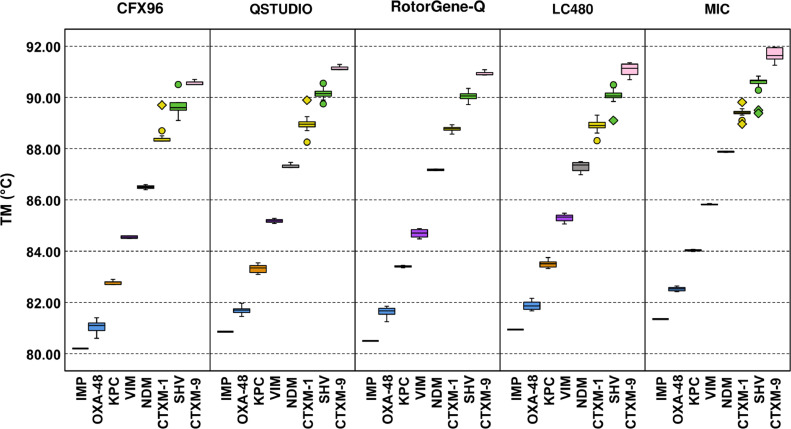
Melting temperatures (*T*_m_) of the eight amplicons of the air-dried high-resolution melt (HRM) assay run in the CFX96, QuantStudio^TM^ 5 (QSTUDIO), Rotor-Gene® Q (RotorGene-Q), LightCycler® 480 (LC48) and Magnetic Induction Cycler (Mic). The whiskers show the maximum and minimum values, with the exception of outliers (circles) and extremes (rhombus).

The amplicon *T*_m_ (°C) shifted across platforms (Fig. 2) and ranged from ±0.013–±0.99°C for *bla*_CTX-M-1_, ±0.07–1.09°C for *bla*_CTX-M-9_, ±0.08–1.15°C for *bla*_IMP_, ±0.02–1.26°C for *bla*_KPC_, ±0.01–1.38°C for *bla*_NDM_, ±0.19–1.5°C for *bla*_OXA-48-like_, ±0.08–0.94°C for *bla*_SHV_ and ±0.12–1.27°C for *bla*_VIM_ depending on the platform used. The *T*_m_ differences within the same peak and neighbouring peaks is shown in Tables 4a and 4b for each of the platforms. The *T*_m_ difference was not statistically significant for any of the platforms for either the type of peak, peaks within the same cluster (*P* = 0.318) and neighbouring clusters (*P* = 1.00).

**Table 4:** Variability in melting temperature (*T*_m_) within the same and between neighbouring clusters obtained in the validated platforms

(a) Standard deviation (S.D.) of the *T*_m_ within the same cluster					
	S.D of the *T*_m_ within the same cluster (±°C)				
	CFX96	LightCycler® 480	Mic	QuantStudio^TM^ 5	Rotor-Gene® Q
*bla*_CTX-M-1_	0.27	0.19	0.15	0.25	0.08
*bla*_CTX-M-9_	0.09	0.25	0.28	0.11	0.08
*bla*_NDM_	0.33	0.20	0.31	0.15	0.14
*bla*_SHV_	0.08	0.14	0.03	0.17	0.03
*bla*_KPC_	0.26	0.19	0.07	0.16	0.20
*bla*_OXA-48-like_	0.06	0.18	0.02	0.10	0.18
*bla*_VIM_	0.27	0.19	0.15	0.25	0.08

(b) Mean difference in *T*_m_ within neighbouring clusters					
	Mean difference in *T*_m_ within neighbouring clusters (°C)				
	CFX96	LightCycler® 480	Mic	QuantStudio^TM^ 5	Rotor-Gene® Q
*bla*_OXA-48-like_ & *bla*_IMP_	0.83	0.95	1.18	0.84	1.12
*bla*_KPC_ & *bla*_OXA-48-like_	1.74	1.62	1.51	1.62	1.78
*bla*_VIM_ & *bla*_KPC_	1.78	1.80	1.79	1.86	1.29
*bla*_NDM_ & *bla*_VIM_	1.95	2.00	2.05	2.14	2.48
*bla*_CTX-M-1_ & *bla*_NDM_	1.91	1.61	1.52	1.66	1.60
*bla*_SHV_ & *bla*_CTX-M-1_	1.20	1.15	1.14	1.16	1.27
*bla*_CTX-M-9_ & *bla*_SHV_	0.96	1.02	1.11	1.01	0.88

### 3.4 Limit of detection (LOD)

The LOD was 11.5, 102 and 960 CFU/reaction using DNeasy Kit and 2.3, 20.4 and 192 CFU/reaction by the boilate method for isolates carrying the *bla*_CTX-M-1_, *bla*_SHV_ and both *bla*_CTX-M-1_ and *bla*_SHV_ genes, respectively.

### 3.5 Stability upon different storage conditions

**Fig. 3:**
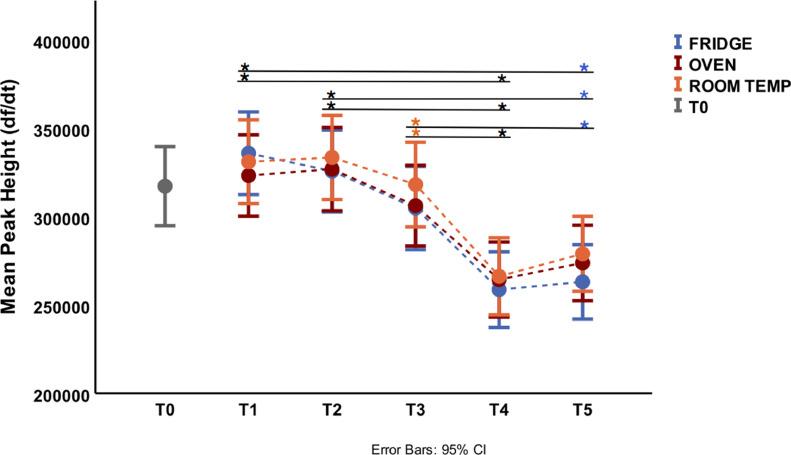
Plate mean fluoresce peak height at the beginning of study (T0) and after 1 week (T1), 2 weeks (T2), 1 month (T3), 3 months (T4) and 8 months (T5) under fridge storage (6.2 ± 0.9°C), at room temperature (20.4 ± 0.7°C) and in an oven (29.7 ± 1.4°C). The colour of asterisks indicates which storage conditions were statistically different between time points: blue (fridge), orange (room temperature), red (oven) and black (all temperature conditions). CI, confidence interval.

The effect of storage time and temperature on the air-dried HRM assay was assessed by analysing the plate mean fluorescence peak height and amplification of isolates, including at the LOD dilution. The average temperature for room, fridge and oven storage was 20.4 ± 0.7°C, 6.2 ± 0.9°C and 29.7 ± 1.4°C, respectively, and the humidity of the room was 36.5 ± 9.34%. Overall, room temperature was the best storage condition compared with the fridge and oven. The difference in mean fluoresce peak hight was not statistically significant within the same time point but was statistically significant between different time points (Fig. 3). The peak height started decreasing after storage time T3 for room and oven storage and at T2 for fridge storage (Fig. 3). None the less, the difference in mean peak height produced with the air-dried HRM assay stored at time T3 (1 month) was not statistically significantly different to that produced at T0, T1 and T2 at all storage conditions. The air-dried HRM assay recovered at T4 and T5 (fridge only) produced significantly lower peak heights compared with T1, T2 and T3 (room temperature only). The mean peak height produced with the air-dried HRM assay stored at time T5 at room temperature was comparable with all time points at all storage conditions and timepoints except at T1 for fridge storage (Fig. 3).

**Fig. 4:**
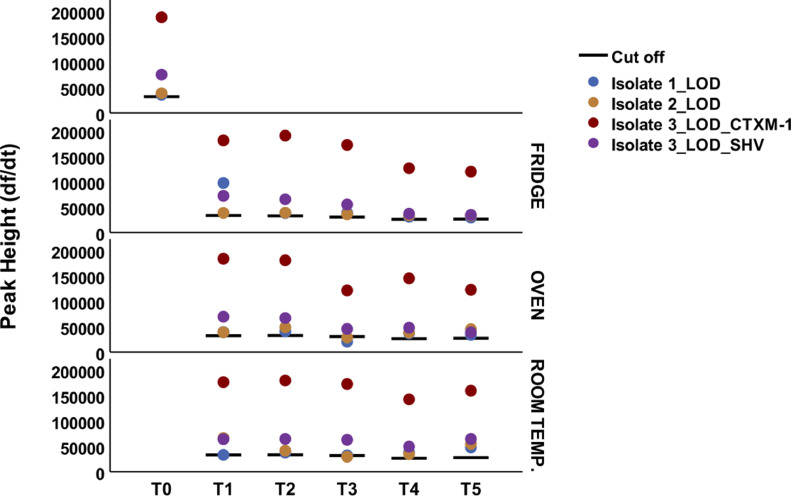
Peak height of isolate 1 (*bla*_CTX-M-1_-positive), isolate 2 (*bla*_SHV_-positive) and isolate 3 (*bla*_CTX-M-1_- and *bla*_SHV_-positive) at the limit of detection (LOD) dilution at different timepoints and storage conditions.

Isolate 1 was negative at the LOD dilution at T3 under oven storage; isolate 2 was negative at the LOD dilution at T3 under room temperature and oven storage; and isolate 3 was positive in all runs tested (Fig. 4). Of the 89 isolates tested, 100% were positive for all markers at all storage times and conditions, except for one sample that had one of three marker peaks below the cut-off (*bla*_NDM_) at T4 fridge storage (data not shown).

## 4 Discussion

In this study, we evaluated the performance of a dry-format 8-plex HRM PCR assay to detect ESBL and carbapenemase genes. The assay showed high sensitivity, specificity and measures of agreement for all markers compared with the reference tests. In addition, the drying process did not result in loss of performance, with all of the resistance genes of the 89 clinical isolates correctly classified after 6 months of storage.

The dry format of the assay overcomes key real-world challenges relating to transport, storage and freezing/thawing issues, which can substantially lower the sensitivity of PCR [27,28]. This HRM assay presents several major advantages over fresh qPCR mixes as it is resistant to long periods of storage at relatively warm temperatures (30°C). Additionally, the HRM assay is more economic than fluorescent probe-based assays and has good performance using the boilate extraction method. This would be of particular importance in LMICs where laboratories face insufficient and suboptimal cold chain capacity and scarcity of funds [29].

The air-dried HRM assay mix recovered at T4 and T5 stored in the fridge had lower peak heights than at oven and room temperature. This indicates that fridge storage is the less suitable for this assay than room and oven temperature. However, detection of *bla*_CTX-M-1_ and *bla*_SHV_ was compromised at the LOD dilution in isolates 1 and 2 at T3 for oven and room storage, suggesting that detection of genes at very low concentrations can be compromised after 1 month of storage.

Interpretation of the results via analysis of the melting data can be automated in the systems’ software, which reduces subjectivity and intra-operator variation. The assay would be implementable in laboratories with access to qPCR facilities but otherwise moderate resources, as all that is required is to reconstitute the mix and add template DNA. The level of multiplexing enables detection of the eight major carbapenemase and ESBL gene families in a single tube with a sensitivity and specificity comparable with reference molecular tests. Molecular detection of AMR genes can provide useful epidemiological data and enable the tracking of particular resistance genes at a hospital or national level [30].

Cross-platform validation illustrates a remarkably good performance on all five qPCR systems evaluated (Rotor-Gene® Q, QuantStudio^TM^ 5, CFX96, LightCycler® 480 and Mic), with minimal variation on the peak *T*_m_. The cut-offs, however, required slight adjustment (5% or 10% of the highest peak) to achieve the best performance; nevertheless, this is straightforward correction that is automated for peak calling.

The protocol has some constraints as a 24-h incubation from primary sample to grow the isolates is still required prior to DNA extraction. The assay has not been evaluated using direct clinical samples, but the LOD obtained here indicates sensitivity to be insufficient to detect the low CFU/mL (>1/mL) possible in bacterial bloodstream infections [31,32]. Since an internal amplification control has not been included to maximise sensitivity, the assay should be used with caution on samples that might contain PCR inhibitors such as stool or soil.

Another constraint of the air-dried HRM assay is the limitation to distinguish between narrow-spectrum and extended-spectrum resistance genes. This is particularly important for *bla*_SHV_; however, as many *bla*_SHV_ found in non-*Klebsiella* spp. are ESBLs [33], all *bla*_SHV_ were considered ESBL to maximise the sensitivity of the test. This may overestimate resistance if is not interpretated with knowledge of the local epidemiology.

The overall agreement to predict bacterial phenotypes was strong amongst Enterobacteriaceae isolates but weak in non-Enterobacterial isolates. Thus, we do not recommend use of the assay in non-Enterobacterial isolates. The high discrepancy among non-Enterobacteriaceae isolates can be explained as *Acinetobacter* spp. and *Pseudomonas* spp. have other frequent mechanisms of resistance such as efflux pumps, permeability defects and modification of target sites that are less common in the Enterobacteriaceae family [34,35] as well as chromosomal-mediated AmpC enzymes [36] or *bla*_GES-1_ [37]. A HRM assay for the detection of AmpC enzymes has already been developed [22] and could be easily adapted to a two-tube AmpC and ESBL-Carb air-dried HRM assay using the methodology detailed here.

Other reasons for phenotype–genotype mismatches include enzyme modifications that change the spectrum of activity and susceptibility profile [38] as well as isolates with minimum inhibitory concentrations (MICs) close to the breakpoint being incorrectly classified during phenotypic susceptibility testing [39].

To summarise, the air-dried HRM assay rapidly detected ESBL and carbapenemase genes with high specificity and sensitivity and maintained performance after 6 months of storage at room temperature. This 8-plex dry HRM assay was also successfully transferred to five different qPCR platforms, indicating that can be reliably implemented in many laboratories. The assay can become a useful tool for AMR diagnosis and surveillance.

## Appendix Supplementary materials

Image, application 1
